# Design and Implementation of a Culturally-Tailored Randomized Pilot Trial: Puerto Rican Optimized Mediterranean-Like Diet

**DOI:** 10.1016/j.cdnut.2022.100022

**Published:** 2022-12-23

**Authors:** Josiemer Mattei, Claudia B. Díaz-Alvarez, Charmaine Alfonso, H June O’Neill, Carlos F. Ríos-Bedoya, Vasanti S. Malik, Filipa Godoy-Vitorino, Chao Cheng, Donna Spiegelman, Walter C. Willett, Frank B. Hu, José F. Rodríguez-Orengo

**Affiliations:** 1 Department of Nutrition, Harvard TH Chan School of Public Health, Boston, MA, USA; 2 FDI Clinical Research, San Juan, PR, USA; 3 College of Nutritionists and Dietitians of Puerto Rico, San Juan, PR, USA; 4 School of Health Sciences, Ana G. Méndez University, Gurabo Campus, Gurabo, PR, USA; 5 McLaren Health Care, Graduate Medical Education, Grand Blanc, MI, USA; 6 Department of Nutritional Sciences, University of Toronto, Ontario, Canada; 7 Department of Microbiology, School of Medicine, University of Puerto Rico Medical Sciences Campus, San Juan, PR, USA; 8 Department of Biostatistics and Center for Methods in Implementation and Prevention Science, Yale School of Public Health, New Haven, CT, USA; 9 Department of Biochemistry, School of Medicine, University of Puerto Rico Medical Sciences Campus, San Juan, PR, USA

**Keywords:** mediterranean diet, Puerto Rico, cardiometabolic health, dietary intervention, cultural adaptation, legumes, plant-based oils, clinical trials

## Abstract

**Background:**

Adhering to a Mediterranean Diet (MedDiet) is associated with a healthier cardiometabolic profile. However, there are limited studies on the MedDiet benefits for non-Mediterranean racial/ethnic minorities, for whom this diet may be unfamiliar and inaccessible and who have a high risk of chronic diseases.

**Objectives:**

To describe the study design of a pilot trial testing the efficacy of a MedDiet-like tailored to adults in Puerto Rico (PR).

**Methods:**

The Puerto Rican Optimized Mediterranean-like Diet (PROMED) was a single-site 4-mo parallel two-arm randomized pilot trial among a projected 50 free-living adults (25–65 y) living in PR with at least two cardiometabolic risk factors (clinicaltrials.gov registration #NCT03975556). The intervention group received 1 individual nutritional counseling session on a portion-control culturally-tailored MedDiet. Daily text messages reinforced the counseling content for 2 mo, and we supplied legumes and vegetable oils. Participants in the control group received cooking utensils and one standard portion-control nutritional counseling session that was reinforced with daily texts for 2 mo. Text messages for each group were repeated for two more months. Outcome measures were assessed at baseline, 2 and 4 m. The primary outcome was a composite cardiometabolic improvement score; secondary outcomes included individual cardiometabolic factors; dietary intake, behaviors, and satisfaction; psychosocial factors; and the gut microbiome.

**Results:**

PROMED was designed to be culturally appropriate, acceptable, accessible, and feasible for adults in PR. Strengths of the study include applying deep-structure cultural components, easing structural barriers, and representing a real-life setting. Limitations include difficulty with blinding and with monitoring adherence, and reduced timing and sample size. The COVID-19 pandemic influenced implementation, warranting replication.

**Conclusions:**

If PROMED is proven efficacious in improving cardiometabolic health and diet quality, the findings would strengthen the evidence on the healthfulness of a culturally-appropriate MedDiet and support its wider implementation in clinical and population-wide disease-prevention programs.

## Introduction

Cardiometabolic health may be maintained by adhering to a healthy dietary pattern. A well-studied pattern is the Mediterranean diet (MedDiet) characterized by mainly consuming plant-based foods, including fiber-rich cereals, fruits, vegetables, olives, nuts, and seeds; consuming legumes, nuts, fish, and eggs as primary sources of protein; using olive oil; and consuming dairy products in moderation [[1], [2], [3]]. Alcoholic beverages—predominantly wine—are permitted in moderation [[1], [2], [3]]. Multiple studies have shown that adhering to a MedDiet is associated with a lower risk of CVD and cerebrovascular disease, several types of cancer, type 2 diabetes (T2D) and glycemic dysregulation, and better cognitive function and mental health [[4], [5], [6], [7]].

One seminal clinical trial testing the efficacy of the MedDiet on cardiometabolic risk factors was conducted in Spain between 2003 and 2012. The Prevención con Dieta Mediterránea (PREDIMED) was a multicenter, randomized controlled clinical trial that aimed to assess the effects of the MedDiet on primary prevention of CVD [8]. The study randomly assigned participants with a high risk of CVD a MedDiet supplemented with either extra virgin olive oil or mixed nuts (that is, walnuts, hazelnuts, almonds) or a reduced-fat control diet [9]. The trial showed benefits of the MedDiet within 3 mo, with significant reductions in plasma glucose, systolic blood pressure, and the ratio of total cholesterol-to-high-density lipoprotein cholesterol (HDL-C) in both the olive oil group and the nuts group, and C-reactive protein (CRP) in the olive oil group, compared with the control diet [10]. Compared with the control diet, long-term data from PREDIMED showed lower blood pressure and reduced risk of major cardiovascular events and T2D for participants in the olive oil or nuts groups [[11], [12], [13]]. The health benefits of a MedDiet have been linked to beneficial changes to the gut microbiome and its metabolites [14].

PREDIMED’s use of olive oil and nuts responds to their status as highly consumed traditional foods in countries bordering the Mediterranean Sea [[1], [2], [3]] and their high content of healthy fatty acids and antioxidants. However, studies on the feasibility and benefits of the MedDiet for non-Mediterranean cultures are limited [15, 16]. This limitation is salient for marginalized and underserved populations with a higher risk of chronic diseases. For example, individuals living in the United States (US) territory of Puerto Rico (PR) have high prevalence of cardiometabolic conditions, including an estimated 16%–26% of adults with T2D, 40% with hypertension, and 68% classified with overweight or obesity [[17], [18], [19]]. Adults in PR have unhealthy dietary patterns characterized by low intake of fruit, vegetables, and whole grains and a high intake of sweets and sugary beverages [20]. Observational studies among Puerto Rican adults suggest that following a Mediterranean-like diet comprised of traditional foods from PR is associated with lower abdominal obesity and body mass index (BMI), less insulin resistance, reduced inflammation, and improved cognitive function [[21], [22], [23]]. Yet, adherence to a MedDiet-like and its potential translation into health benefits have not been tested in Puerto Ricans.

Multiple agencies and committees, including the US Dietary Guidelines for Americans, the American Heart Association, and the World Health Organization, advocate for including nutrient-dense food and beverage choices that reflect personal and ethnic or cultural preferences [[24], [25], [26]]. These calls-to-action highlight the need for culturally-tailored programs that improve upon the existing evidence, leverage available food systems, and value cultural traditions of diverse communities. Ethnically-tailored interventions can be more effective than general dietary messages [[27], [28], [29]]. Thus, reporting the details of a cultural adaptation is essential to inform future processes in other populations. This report describes the study design of a pilot trial that aimed to examine the efficacy of a MedDiet-like tailored to adults in PR by using culturally appropriate foods and strategies for reducing cardiometabolic risk.

## Study Protocols

### Study design

**FIGURE 1:**
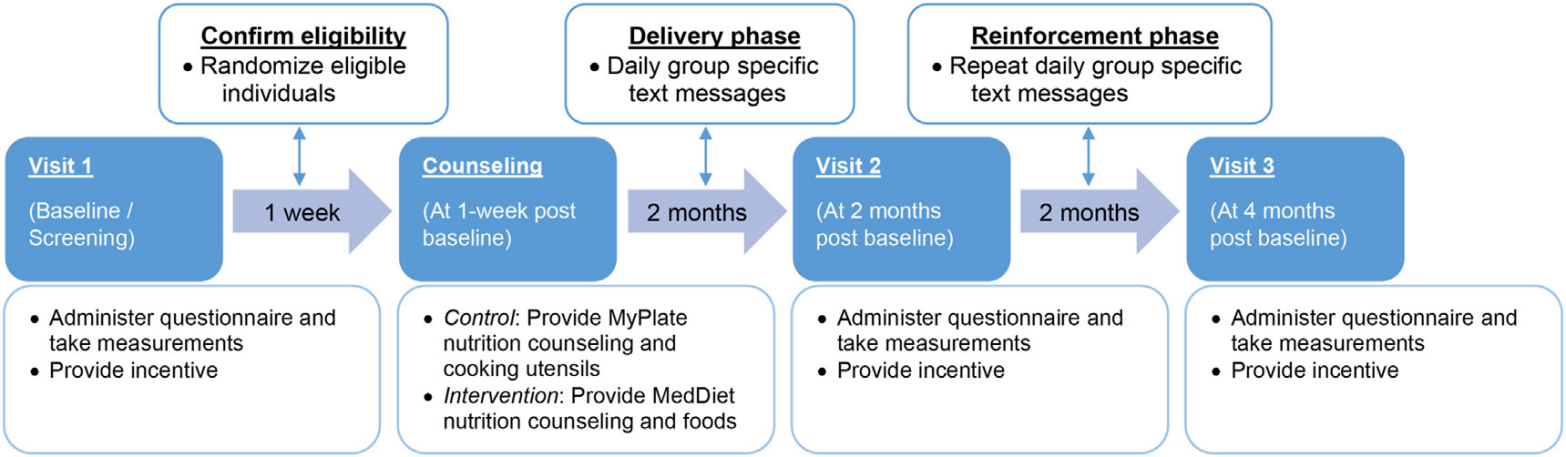
PROMED protocol flowchart. PROMED, Puerto Rican Optimized Mediterranean-like Diet.

The Puerto Rican Optimized Mediterranean-like Diet (PROMED) was a single-site 2-mo parallel two-arm randomized pilot and feasibility trial. The design included an in-person baseline nutritional counseling session among free-living adults (25–65 y) living in PR with at least two cardiometabolic risk factors, followed by text messages with content from the counseling session sent daily for 2 mo (delivery phase) and the same texts repeated daily for two more months (reinforcement phase) (Figure 1). Eligible participants in both the control and intervention groups were asked to attend a baseline, 2- and 4-mo visit for clinical measurements and questionnaires. One week after the baseline visit, the intervention group received one 1-h nutritional counseling session for a portion-control MedDiet-like tailored to adults in PR and a supply of vegetable oil and legumes to last for the first 2 mo. The control group received one 1-h nutritional counseling session for a standard portion-control diet and cooking utensils. A duration of 4 mo for the study was selected, based on early results of the PREDIMED trial that reported significant cardiometabolic improvements at 3 mo [10].

All data collection and procedures were conducted at FDI Clinical Research (FDI), an independent research center and medical care facility in San Juan, PR. FDI is associated with multiple research and clinical institutions and a network of primary care clinics across the archipelago of PR to facilitate recruitment. The Institutional Review Boards of Harvard TH Chan School of Public Health (protocol # IRB19-0184) and Ponce Health Sciences University in PR (protocol #1905013193A001) approved this study. The study was registered at clinicaltrials.gov NCT03975556. The study was conducted from July 2019 to June 2021. The SPIRIT reporting guidelines on study methodology only (no results) were used [30].

### Primary and secondary outcomes

Similar to a previous study [31], the primary outcome of PROMED was a composite cardiometabolic improvement score representing the summed number of improved cardiometabolic risk factors out of the 10 factors that were part of the eligibility criteria; the score has a plausible range of 0–10. Improvement was defined by clinically-meaningful decreases of *1*) waist circumference ≥ 1 cm; *2*) BMI ≥ 1 kg/m^2^; *3*) systolic blood pressure ≥ 5 mmHg; *4*) diastolic blood pressure ≥ 5 mmHg; *5*) plasma glucose ≥ 1 mg/dL; *6*) hemoglobin A1c ≥ 0.5%; *7*) plasma triglycerides ≥10 mg/dL; *8*) total cholesterol ≥ 10 mg/dL; and *9*) LDL-C ≥ 10 mg/dL; or an increase of [8] HDL-C ≥ 5 mg/dL [[32], [33], [34], [35], [36], [37], [38], [39], [40]]. A composite score was selected to account for baseline variability in cardiometabolic risk profiles. Furthermore, the selected markers are routinely measured in standard laboratory tests, and the predefined improvements are clinically meaningful in reducing the risk of chronic disease, facilitating the potential for clinical translation.

Secondary outcomes included changes in the individual cardiometabolic risk factors plus weight, dietary intake, overall diet quality, diet satisfaction, dietary behaviors, and psychosocial factors (e.g., depressive symptoms, stress). An exploratory aim was conducted to assess changes in the gut microbiome profile, based on previous literature suggesting associations between a MedDiet, legumes and oils, and gut microbiota composition and microbial functions [[41], [42], [43], [44]].

### Recruitment

Recruitment was done by posting flyers and posters in the main areas of the partner clinics; direct contact with patients and visitors to the partner clinics, where trained research assistants visited the waiting room and lobbies to distribute flyers to all individuals present and answer questions about the study; advertising in community locations and events (e.g.,., health fairs, festivals, supermarkets, shopping centers, parking lots) and social media (Facebook); and [4] through referrals from other participants.

Interested individuals contacted the study and were screened for potential eligibility using a phone-administered screening questionnaire. Potential eligibility was initially assessed based on *1*) age 25–65 y at the time of enrollment; *2*) noninstitutionalized (that is, not committed to an institution at the time of enrollment; such as prison, in-patient hospital, or treatment center); *3*) living in PR at the time of recruitment and for at least the previous year and not planning to move from PR within the next 6 mo; *4*) able to answer questions without assistance (that is, no severe psychological disorder or mental and/or physical disability that would prevent completion of interview); *5*) having a cellphone with the capacity to receive text messages and being willing to receive daily text messages from the study; and *6*) self-reporting at least two of five cardiometabolic conditions (elevated waist circumference, overweight or obesity, prediabetes, hypertension or use of blood pressure medication, dyslipidemia (high triglycerides or high LDL-C or low HCL-C or use of lipid-lowering medication).

If eligibility was probable based on these criteria, the individual was invited to a baseline visit where the research assistants used a scripted guide with clear, concise points to explain the study’s purpose, risks, and benefits, and any answer questions. Participants had the option of having a health proxy in the room during this process. Written informed consent to participate was obtained, including authorization to publish anonymized results as aggregated data. At the baseline visit, interviews and clinical and laboratory measurements were conducted to confirm the cardiometabolic conditions for eligibility, defined as waist circumference ≥94 cm in men or ≥80 cm in women; BMI ≥25 kg/m^2^; measured high systolic blood pressure (≥120 mmHg) or diastolic blood pressure (≥80 mmHg) (or use of blood pressure medication); measured prediabetes (fasting plasma glucose of 100 to 125 mg/dL (5.6 to 6.9 mmol/L) or hemoglobin A1C of 5.7 to 6.4% (39 to 47 mmol/mol); and measured total cholesterol ≥200 mg/dL or triglycerides ≥150 mg/dL or LDL-C ≥130 mg/dL or HDL-C <40 mg/dL, or use of lipid-lowering agents.

Individuals were excluded from the study for several reasons: *1*) self-reported physician-diagnosed type 1 or type 2 diabetes or use of diabetes medication or had a measured fasting plasma glucose ≥126 mg/dL (7.0 mmol/L) or hemoglobin A1C ≥ 6.5% (48 mmol/mol) at the baseline visit; *2*) reported current pregnancy; *3*) reported condition that would influence eating behaviors or nutritional status (e.g., Chron disease, inflammatory bowel disease, celiac disease, liver disease, kidney disease, cancer, diverticulitis, nut allergy, severe food allergies, or aversions); and *4*) intolerance or allergies to legumes (that is, beans and nuts) or vegetable oils (that is, soybean, olive, canola). These exclusion criteria were asked at subsequent visits, and individuals were withdrawn from the study if they answered affirmatively to any of these questions.

The blood test results of potentially-eligible individuals were received within 1 wk of the baseline visit. The values were used to confirm eligibility. If a person was deemed ineligible based on measured laboratory values, the individual was immediately informed and withdrawn from the study. Eligible participants were invited to complete the study examinations and surveys at FDI.

### Randomization and data collection

Confirmed, eligible, and consented participants were randomly assigned to one of the two study groups (control or intervention) in a 1:1 allocation ratio. These participants were randomized using sex stratification (40% males and 60% females) and permuted blocks with random block sizes. For this randomization scheme, a sequence of block sizes was randomly generated in which allowable blocks were a random mix of sizes 2 or 4 participants. Thus, half of the participants within each block were randomly assigned to the control group and the other half to the intervention group.

Trained research assistants conducted all visits in a single private room at FDI. Research assistants were bilingual (English and Spanish) and trained in culturally sensitive interviewing, clinical protocols, data collection, data entry, confidentiality, and safety measures. Each clinical visit lasted ∼2 h. The counseling session lasted 1 h. Participants were compensated for their time up to $150, such that they received $50 at each of the three examination visits, with no monetary incentive at the counseling session. At the counseling session, participants received a bag with food (intervention group) or cooking utensils (control group). A light, healthy snack, and water were provided after each visit.

Participants were allowed to pause or terminate the interviews at any time. In such case, the participant was contacted up to five times within the subsequent 14 d to complete the pending questionnaires or measurements. Participants remained enrolled in the study for its duration (4 mo) unless they actively left the study, had a new ineligibility factor, or lost contact. All interview questions and outcome measures at 2  and 4 mo were assessed using the same protocols at baseline.

### *Questionnaires*

The research assistant administered a questionnaire that asked about age, sex-at-birth, household income, educational attainment, employment, household composition, menopausal status, healthcare services, medical diagnoses, family history of major diseases, food security and assistance, smoking status, alcohol use, sleep quantity and quality, moderate and vigorous physical activity, and satisfaction and engagement with the program. A perceived stress scale captured feelings of stress within the previous month [[45], [46], [47]]. Depressive symptomatology was captured using the Center for Epidemiologic Studies Depression Scale [[48], [49], [50]]. The University of California-Los Angeles Loneliness 3-item scale measured overall loneliness [51]. The Interpersonal Support Evaluation List-12 assessed social support in three domains: appraisal, belonging, and tangible support [52, 53].

### *Dietary assessments and measures of adherence*

Diet was assessed at the clinic visits using the Spanish version of a brief diet quality index with questions on frequency and amount of intake in the past month of 18 major Mediterranean food groups, after minor linguistic and cultural adaptations [54]. Daily intake of one serving (1 s/d) of bread, vegetable, fruit, yogurt/milk, rice/pasta, olive/corn/sunflower oil, alcoholic beverages, and breakfast cereal was assessed. Three points were given for intake >1 s/d, two points for 1 s/d, and one point for <1 s/d. Weekly servings of meat, sausage, cheese, pastry/sweet, butter/lard, other oils, or fast foods were assessed as 1/wk, 4–6/wks, or >6/wks; these frequencies were given three points, two points, and one point, respectively. Fish, legumes, and nuts were assessed as >3/wks, 2–3/wks, and <2/wks, which were scored three points, 2 points, and two points, respectively. Food group points were summed to generate a total score ranging from 18 (no adherence to the MedDiet) to 54 (optimal adherence). The index was highly correlated with 24-h recalls (*r* = 0.61) and was positively associated with intake of various nutrients.

A short food frequency questionnaire captured the frequency of consumption of 33 major food groups during the previous month. These questionnaires were supplemented with questions about specific types and amounts of legumes and vegetable oils intake during the previous month to gauge further adherence. Compliance to the intervention recommendations was defined as reporting consumption of one serving of vegetable oil intake >1 times/d and of legumes >3 times/wk. Compliance to the control recommendations was defined as reporting consumption of one serving of meat (lean beef, pork, or poultry) >6 times/wk. These foods were emphasized in the corresponding group’s counseling session. The study satisfaction and engagement section also asked about self-perceived adherence to the program.

Diet satisfaction was measured with the 45-item Diet Satisfaction Questionnaire that includes seven scales to assess healthy lifestyle, cost, convenience, family dynamics, preoccupation with food, negative aspects, and planning and preparation [55]. Emotional and restrictive eating behaviors were captured with the Three-Factor Eating Questionnaire [56]. The frequency of food behaviors (that is, cooking and eating practice, foods consumed away from home, nutrition awareness, and supplements used) was asked [17, 57].

### *Anthropometry and blood pressure*

Participants were instructed to wear light clothing and remove any outerwear, stand with feet close together, arms at the side, and body weight evenly distributed, and relax and breathe normally; measurements were taken at the end of a normal expiration. The research assistant measured the participant’s waist and hip circumference to the nearest 0.1 cm using a stretch-resistant measuring tape following standard protocols, that is, waist at the midpoint between the lower margin of the least palpable rib and the top of the iliac crest; hip around the widest portion of the buttocks, with the tape parallel to the floor [58, 59].

Weight was measured using a Detecto SLIMTALKXL scale to the nearest 0.1 kg (Cardinal/Detecto). Height was measured using a Seca 213 stadiometer to the nearest 1 mm (Seca). With the participant seated for 5 min and feet flat on the floor, blood pressure and pulse were measured with a clinically-validated Omron 10 Series Upper Arm Monitor Model BP7450 (OMRON Healthcare) at either arm, at the beginning, midpoint, and end of the visit [60, 61]. All measurements were repeated thrice; the averaged values were used.

### *Laboratory measurements*

Participants were asked to refrain from consuming food or beverages other than water for 12 h before the scheduled appointment; fasting status was confirmed by the participant before the blood draw. Fasting blood samples were obtained via venipuncture by a licensed nurse following standardized protocols and safety procedures; sampling tubes for glucose analysis contained antiglycolytic agents. Plasma was separated within 2 h of the blood draw at FDI in a refrigerated centrifuge. Samples were refrigerated or frozen until collected for laboratory analysis at the CLIA-approved Toledo Laboratory. A blood sample was stored at −80°C for potential use in future studies for participants who consented to this part of the study.

Blood samples were analyzed in the Alinity-c Systems (Abbott) for lipid panel using enzymatic reagents for total cholesterol, the glycerol phosphate oxidase method for plasma triglycerides, an accelerator selective detergent for HDL-C, the hexokinase method for plasma glucose, chemiluminescent microparticle immunoassay for insulin, and a comprehensive metabolic panel for liver and renal biomarkers (e.g., alanine aminotransferase, alkaline phosphatase, aspartate aminotransferase, creatinine). The Friedewald formula was used to estimate LDL-C; VLDL-C was calculated as triglycerides/5.00. The homeostasis model assessment of insulin resistance (HOMA-IR) was calculated from paired fasting glucose and insulin values using the HOMA calculator version 2.2.3 [62]. High-sensitivity CRP was measured using immunoturbidimetric MULTIGENT CRP Vario assay in the Abbott Architect system. Hemoglobin A1C was analyzed using non-porous ion-exchange high-performance liquid chromatography in a Tosoh Automated Glycohemoglobin Analyzer G8 (Tosoh Bioscience). Platelet count was obtained by Sysmex XN-1000 multi-parameter automated hematology analyzer (Sysmex Corporation).

### *Fecal sample and microbiome analysis*

On the first visit, participants were given a kit containing a clean plastic biohazard bag and a 30 mL falcon tube container to collect the fecal sample at home. Instructions were provided for proper collection and handling on the morning of the next visit (preferably) or the day before. Briefly, the stool was to be collected with a collection hat or strong paper to avoid contamination with urine. The stool was scraped with the collection stick or scoop and inserted into the falcon tube until sufficient stool was obtained (about 0.8 inches or 21 mm). The participant placed the specimen in the biohazard bag, documented the date and time of collection on the bag, and placed the bag in a −20°C freezer (standard home freezer temperature) until the next visit, when it was immediately stored at −80°C. Fecal samples were packaged in dry ice and directly transferred for microbiome profile analysis at the Microbiome Lab of the University of Puerto Rico Medical Sciences Campus. Genomic DNA was extracted with the QIAgen Powersoil Kit [63]. The V4 hypervariable region of the 16S ribosomal RNA marker gene (∼291bp) was amplified using universal bacterial primers following standards from the Earth Microbiome Project [64]. Sequences of the 16S gene V4 region were deposited in the QIITA database to be analyzed for standard human microbiome methods using QIIME2 and then made publicly available [65]. The same protocols as the American Gut Project and Human Microbiome Project Phase were used for fully comparable data [66, 67].

### Intervention

**FIGURE 2:**
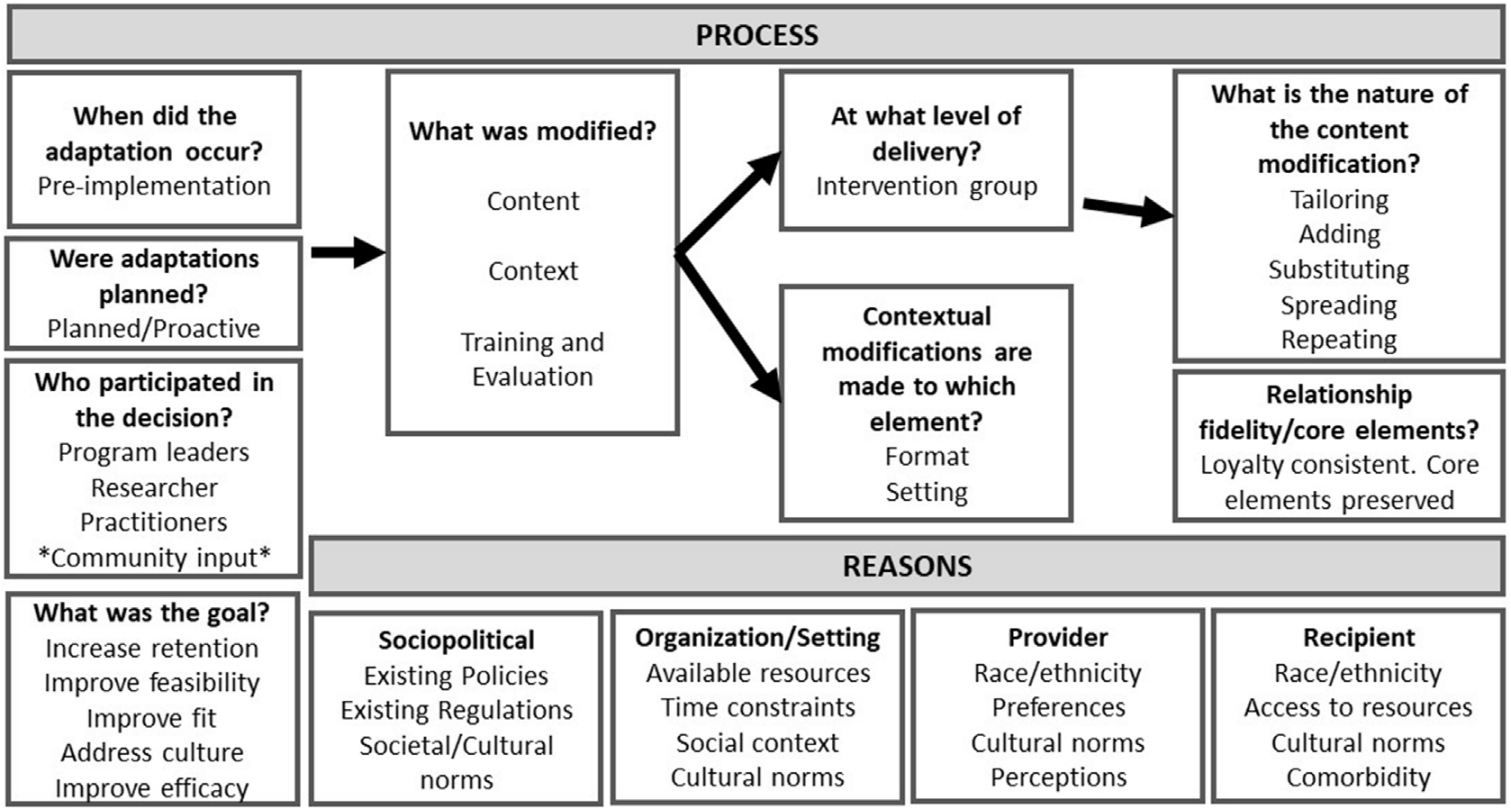
Factors for the cultural adaptation process of PROMED following the Framework for Reporting Adaptations and Modifications-based Implementation Strategies (with permission from Wiltsey–Stirman). PROMED, Puerto Rican Optimized Mediterranean-like Diet.

The Framework for Reporting Adaptations and Modifications-based Implementation Strategies was used to depict the cultural adaptation process (Figure 2) [68]. Several sociopolitical, organizational, provider-based, and recipient-based reasons to adapt the intervention were identified. Thus, the program’s adaptation was planned proactively (before implementation) with input from PR community members during formative research and decisions made by program leaders, researchers, and practitioners. Modifications included tailoring, adding, substituting, spreading, and repeating content (e.g., message), context (e.g., foods, strategies), or training/evaluation (e.g., surveys), as well as format and setting, of the original materials. The adaptation remained mostly consistent to the core elements of healthy eating counseling. The goals of the adaptation were to increase retention, address cultural competency, and improve feasibility, fit, and efficacy in the intended population.

### *Cultural tailoring: food*

PROMED was culturally tailored by replacing PREDIMED’s intervention foods with traditional Puerto Rican foods. PREDIMED supplemented a MedDiet with olive oil and mixed nuts. In PR, the top consumed oils are canola, corn, olive, sesame, or soybean oils; a ranking of foods within a MedDiet score in PR adults did not identify olive oil as the primary type of monounsaturated fatty acids (MUFA) source [[20], [21], [22]]. PROMED thus provided olive oil, given its cardiometabolic benefits [69, 70], and a blend of canola and soybean oils. A literature review documented statistically and clinically significant reductions in total cholesterol and LDL-C and improved insulin sensitivity from consuming canola oil compared with other dietary fat sources [71]. Clinical and epidemiologic studies suggest that consuming soybean oil can lower blood cholesterol concentration when replacing dietary saturated fats [72]. Providing a blend of oils was also important to diversify the ratios of MUFAs to polyunsaturated fatty acids (PUFAs) and the specific types of fatty acids [73]. For example, olive oil and canola oil mainly contain MUFAs, but olive oil is >70% oleic acid and just 0.6% alpha-linolenic acid (ALA), an omega-3 fatty acid, whereas canola oil is 60% oleic acid and contains 10% ALA. Soybean oil is also high in ALA (9%), with the rest of its composition being other PUFAs.

Legumes were selected instead of mixed nuts because beans are a main staple food of the Puerto Rican diet. However, studies have shown reduced consumption during a recent nutrition transition [74, 75]. A ranking of the percent contribution to the total energy of foods consumed by adults in PR showed that legumes (specifically, pinto, black, kidney, lima beans, chickpeas, and green peas) ranked higher than nuts (specifically, almonds, cashews, peanuts, pecans, pine nuts, sunflower seeds, walnuts, pistachios, and peanut butter), with a total energy contribution of 3.14% (or 0.63 serv/d) vs. 1.31% (or 0.23 serv/d), respectively [20]. Additionally, an analysis of foods contributing to the “legumes and nuts” component of a MedDiet score in Puerto Ricans identified beans (that is, pinto, white, kidney, black, and lima) and peanuts (a legume) as top contributors [21, 22]. The selection was further supported by research showing that legume consumption reduces the risk of T2D risk factors, including abdominal adiposity and metabolic syndrome [[76], [77], [78]], given their high content in essential amino acids, folate, soluble fiber, copper, magnesium, iron, potassium, calcium, zinc, and ALA [79]. Peanuts were included in the legumes component to enhance the intake of polyphenols, micronutrients, and linoleic acid (an omega-6 fatty acid). Furthermore, in small clinical trials, peanuts have decreased glucose concentration without weight increases [80, 81]. Consuming peanuts has a similar effect as almonds on improving fasting and postprandial blood glucose among patients with T2D [82], and may reverse metabolic syndrome when replacing refined grain snack bars among adults in China [83].

On the day of the counseling session, participants in the intervention group were given sufficient dry and/or canned beans or peanuts to consume a recommended four portions of legumes weekly (that is, 1 portion = ½ cup of beans or ¼ ounce of peanuts) and four servings of 1 tsp each of oils daily (2 tsp for direct consumption such as dressings and 2 tsp for food preparation) (Table 1). Thus, for 2 mo, 16 portions of ½ cup of legumes and 224 (37 ounces) portions of 1 tsp of vegetable oils were needed. Participants were given an excess of food (that is, 45 portions of legumes and 444 portions of oils), as these are usually used for preparing household meals. The portions were based on recommendations by the American Heart Association, the USDA Dietary Guidelines, and the PR Dietary Guidelines [[84], [85], [86], [87]] Participants were not given any other guidance on the timing of consumption or maximal intake amounts and could thus consume these foods *ad libitum* within the recommended portion. At the 2-mo visit, no more food was given. Participants kept any remaining food not consumed by the 2-mo visit.

**TABLE 1:** Characteristics of the legumes and vegetable oils given to participants in the intervention group of PROMED

Product	Quantity	Portion size	Portions provided for 2 mo	Selected nutrient profile (per portion)	Estimated total cost∗
Legumes					
Low-sodium canned pink beans	2 cans (15.5 oz each)	½ cup	8	Fiber: 2.2 g Fe: 0.6 mg K: 118 mg Mg: 19 mg	$1.78
Low-sodium canned garbanzo beans	2 cans (15.5 oz each)	½ cup	8	Fiber: 2.2 g Fe: 0.7 mg K: 86 mg Mg: 14 mg	$1.78
Low-sodium canned white beans	2 cans (15.5 oz each)	½ cup	8	Fiber: 2.4 g Fe: 1.4 mg K: 227 mg Mg: 25 mg	$1.78
Dried pigeon peas	1 bag 16 oz	½ cup	6	Fiber: 7.5 g Fe: 2.6 mg K: 696 mg Mg: 92 mg	$1.29
Dried small red beans	1 bag (16 oz)	½ cup	6	Fiber: 12.5 g Fe: 4.1 mg K: 703 mg Mg: 70 mg	$1.29
Unsalted peanuts	1 can (16 oz)	¼ oz	9.1	Fiber: 0.6 g Fe: 0.2 mg K: 47 mg Mg: 12.6 mg MUFA: 1.8 g PUFA: 1.1 g	$4.55
**Vegetable oils**					
Olive Oil	1 bottle (34 oz)	1 tsp or 5 mL	204	MUFA: 14.5 g PUFA: 2.1 g Ratio n6/n-3: 16	$8.15
Vegetable oil mix (blend of soybean and canola oil)	1 bottle (40 oz)	1 tsp or 5 mL	240	Soybean: MUFA: 4.6 g PUFA: 11.5 g Ratio n6/n-3: 6.7 Canola: MUFA: 12.9 PUFA:1.4 g Ratio n6/n-3: 2.2	$3.37

Fe, iron; K, potassium; Mg, magnesium; MUFA, monounsaturated fatty acids; n6/n-3, omega-6 fatty acids/omega-3 fatty acids; PROMED, Puerto Rican Optimized Mediterranean-like Diet; PUFA, polyunsaturated fatty acids. Nutritional data obtained from INCAP [127] and Duboi et al. [128]. ^1^Excludes tax; costs as of May 2019.

Instead of food, participants in the control group received a set of cooking utensils. For both treatment groups, participants were instructed to follow the recommendations at their own pace in no specific order or timing, and to continue their usual lifestyle behaviors, including current physical activity.

### *Cultural tailoring: structural barriers*

Structural barriers were also considered in PROMED’s adaptation, including access, cost, and familiarity and acceptability of foods. Nutritionists and dietitians surveyed in PR considered legumes as a food easy to include in the diet of Puerto Ricans. In contrast, fats and fried foods were deemed challenging to control or decrease, suggesting that a shift toward healthy fats may be feasible rather than a reduced-fat diet [88]. Although olive oil is widely available in PR, in 2019 an ounce of olive oil cost $0.24 vs. $0.08 USD for canola or soybean oil. Similarly, beans are found in all food establishments (including corner stores and even pharmacies and gas stations) in PR and cost about $0.06 to $0.08 USD per ounce in 2019. In contrast, raw nuts are only found in supermarkets or specialty stores, and an ounce of almonds costs $0.78 USD. For the intervention, various beans in low-sodium cans and dried bean bags were chosen based on reported cultural preferences [75]. Locally known brands of legumes and oils available at local food markets were obtained to ensure participants’ familiarity and access to the products.

### *Cultural tailoring: counseling and text messages*

The original material to be adapted (that is, the control group) was the MyPlate for a Healthy PR from the Guidelines for Healthy Eating and Physical Activity for PR, which provides the healthy eating recommendations to the population [87], along with the standard portion-control healthy eating lesson used by nutritionists and dietitians in PR, provided by the College of Nutritionists and Dietitians of PR. The MyPlate for a Healthy PR illustrates five food groups using a place setting (fruit, vegetables and legumes, cereals and starches, dairy, and proteins), plus physical activity recommendations. A sixth group (fats and oils) is mentioned in the educational guide. At the 1-h individual counseling session, participants in the control group received the standard healthy eating counseling with no cultural tailoring. A visual guide was provided as a reference (Supplemental Figure 1).

The adaptation for the materials in the intervention group was guided by published literature and formative research conducted by the study team [17, 75, 88]. The adaptation included deep-structure strategies that have been considered useful and culturally appropriate for healthy eating in this population. Deep-structure factors refer to the embedded cultural norms, beliefs, and values that define the nonvisible traits of a person, versus superficial surface-level factors, and that may increase acceptability, efficacy, and translation of health behavior interventions in communities of diverse cultural backgrounds [89, 90]. For this population, such strategies included personalized counseling, setting short-term goals, making gradual dietary changes (that is, following recommendations at their own pace), setting health-oriented and personal goals without focusing on weight loss or dieting, using visual guides, and showing portion size [88]. The 1-h individual counseling session of the intervention group focused on recommendation for portion-control healthy eating based on the MedDiet. A visual guide provided to the participants (Supplemental Figure 2) showed seven food groups, also using a place setting: vegetable oils and fats, vegetables, cereals, grains and legumes, fruit, proteins (fish, chicken, and turkey; with dairy also mentioned in the educational guide), and water. Advice for alcohol intake and for using herbs and spices as a salt-reducing strategy was provided in the educational guide. These groups were selected to cover the most common MedDiet food groups (fruit, vegetables, cereals, fish, legumes and nuts, meat, sodium, alcohol, healthy fats), in lack of consensus [91]. At the counseling session, messages focused on traditional and local food choices and cooking/eating behaviors, and emphasis was given to incorporate legumes (that is, beans and peanuts) and vegetable oils (that is, olive oil and the vegetable blend of canola and soybean oil) at meals throughout the week.

For both arms, the text messages reflected direct or abridged quotes from the counseling materials. Table 2 summarizes the differences in the recommendations of the control and intervention group, against the dietary intake of adults in PR reported in 2015 [20], by food group. Differences can be noted in the food groups emphasized in each group, with the intervention group receiving specific counseling for grains and legumes intake, but not the control, for which beans were part of proteins. The protein group of the MedDiet incorporated minimally-processed choices of dairy (rather than a separate group, as in the control) and recommended limiting red and processed meat, which were recommended as lean choices in the control. Most portion sizes were equal between groups, although recommended amounts for beans and vegetable oils were higher in the intervention group. Although direct comparisons cannot be made between the 2015 intake from adults in PR and the recommendations in PROMED due to differences in food groupings and components, most of the reported intakes were lower than the recommendations, except for root vegetables, vegetables, herbs, and spices. Intake of dairy, and red and processed meats, were also high.

**TABLE 2:** Summary of dietary recommendations for PROMED participants, by treatment group, compared with reported intake of adults in Puerto Rico

	Control^1^	Intervention	Usual intake in adults in Puerto Rico^2^
Cereals	White rice/pasta = 1/3 cup Brown rice = ½ cup Mashed root vegetables = ½ cup Whole root vegetables—2″ × 2″ Bread = 1 slice Cold cereal = ¾ cup Hot cereal = ½ cup Pancake = 4 inches Cracker = 4 squares Grains = ½ cup ∗Mostly whole; variety of root vegetables	Whole wheat bread = 1 slice Whole wheat cracker = 4 squares Pancake = 4 inches Cold cereal = ¾ cup Hot cereal (oatmeal) = ½ cup White rice/pasta = 1/3 cup Mashed root vegetables = ½ cup Whole root vegetables—2″ × 2″	Rice = 0.53 s/d Root vegetables = 1.1 s/d Refined grains = 0.57 s/d Whole grains = 0.53 s/d Pasta = 0.21 s/d
Fruit	Canned = ½ cup Chopped = 1 cup Whole = 1 medium Juice = 4 oz	Whole = 1 medium Chopped = 1 cup Canned = ½ cup 100% Juice = 4 oz	Fruit (no juice) = 1.13 s/d
Vegetables	Cooked = ½ cup Fresh = 1 cup Salad = 1 cup Juice = 4 oz ∗Choose all colors	Fresh Salad = 1 cup Cooked = ½ cup Juice = 4 oz Canned low sodium = ½ cup	Vegetables (no juice) = 3.34 s/d
Grains and Legumes	NA	Beans = ½ cup Nuts = 6 Pecans = 4 halves Peanuts = 10 Peanut butter = ½ tbsp	Nuts = 0.23 s/d Legumes = 0.63 s/d
Proteins	Fish = 3 oz Poultry = 3 oz Lean meat = 3 oz Eggs = 1–3 / wk Cheese = 1 slice Ham = 1 oz or slice Eggs = 1–3/wk ∗Choose lean, fish, legumes, or seeds	Poultry = 3 oz Fish/seafood = 3 oz Natural cheese = 1 slice Egg = 1 Low- or non-fat milk = 8 oz Greek yogurt = 1 cup ∗Limit red and processed meats	Processed meats = 0.72 s/d Red meats = 0.28 s/d Poultry = 0.32 s/d Fish/seafood = 0.37 s/d Eggs = 0.66 s/d
Dairy	Milk = 8 oz Low-fat yogurt = 1 cup Low-fat non-added sugar ice cream = ½ cup ∗Choose low- or non-fat	NA	Dairy = 1.82 s/d
Fats/oils	Oils = 1 tsp Avocado = 1/8 of a medium-sized Peanut butter = ½ tsp Nuts = 6 Peanuts = 10 Pecans = 4	Olive oil = 1 tsp Corn or vegetable oil = 1 tsp Low-fat dressing = 1 tsp oil = 1 tsp Avocado = 1/8 of a medium-sized Margarine = 1 tsp	Oils = 0.34 s/d
Water	With meals throughout the day	Throughout the day	Water = 3.90 s/d
Alcohol	NA	Not recommended to start. If already consuming: limit <2 men; <1 women	Alcohol = 0.24 s/d
Other	NA	Tips for using herbs and spices instead of salt or commercial adobo Tips to incorporate fats/oils in meals	Herbs and spices = 3.03 s/d
Physical activity	30 mins daily		

NA, not applicable; PROMED, Puerto Rican Optimized Mediterranean-like Diet. Recommendations for control and intervention groups are shown in daily servings unless otherwise noticed. ^1^From Comisión de Alimentación y Nutrición de Puerto Rico, Guía alimentaria para Puerto Rico. [87] ^2^From Mattei et al. [20] reflects reported consumption from 2015. Excludes mixed dishes, soups, and foods consumed in fast food restaurants.

### *Text messages: delivery and reinforcement phases*

After the counseling session, daily text messages were sent to participants as reminders of the information received in the counseling session. Several studies have shown that text-based content delivery for health behaviors interventions is acceptable and effective for Latinos [[92], [93], [94], [95], [96], [97]]. Texts were also chosen because >75% of adults in PR use text messaging [17]. Participants received 1 daily text message throughout the 4 mo of the study comprised of advice received at the counseling session corresponding to arm allocation (Table 3). A total of 56 messages were delivered throughout 2 mo, such that 1 daily text was sent for the first 8 wks (2 mo) during the delivery phase. Then, the same 56 messages were repeated with one daily text for the next 8 wks (2 mo) during the reinforcement phase. The messages were distributed among topics selected to strengthen the nutritional counseling given to each group, including major food components. Texts were sent using the Google Voice messaging system. The nutritionist confirmed receipt of the content of the text messages for each participant. Participants could send messages back through the system anytime during the study. If a participant sent a message with a question or comment, the nutritionist answered the question under the supervision of the research team.

**TABLE 3:** Topics and examples of text messages with content from the nutritional counseling sessions for each treatment group in PROMED

Text Message Topic	Intervention group text example	Control group text example
General information on healthy eating	NA	Choose healthy foods and drinks from the five food groups, including fruits, vegetables, cereals, protein foods such as meat, and dairy products, to obtain all necessary nutrients.
Information on the Mediterranean diet	The Mediterranean diet is based on the most common and healthy food patterns of the countries bordering the Mediterranean Sea. Many options of typical Puerto Rican foods can be consumed within this diet.	NA
Cereals	NA	Cereals and farinaceous contain carbohydrates that provide most of the energy that we need.
Whole grains	To eat more whole grains, substitute a whole grain product for a refined product, such as eating whole grain bread instead of white bread or brown rice instead of white rice. Remember to substitute, instead of adding the whole grain product.	NA
Legumes	Prepare dishes with legumes, such as kidney beans, instead of meat, such as in chili, burritos, salads, soups, dips, or stuffed bananas (“canoes”).	NA
Vegetable oils	Consuming unsaturated fatty acids, such as those found in vegetable oils, has positive effects on health, such as increasing amounts of HDL-cholesterol (the “good cholesterol”).	NA
Practical tips	Seasonal fruits and vegetables are usually cheaper and fresher! There are also low-cost healthy foods within the Mediterranean diet available throughout the year: beans, cabbage, sweet potatoes, fresh or canned low-sodium tomatoes, and bananas.	Eat fresh, frozen, canned or dried fruits instead of cookies, brownies or other sugary sweets.
Servings/Portion control	When consuming root vegetables, remember that the portion is a 2″ × 2″ piece (about the size of a credit card). Choose yucca, yam, taro, or *yautía*, and season with olive oil.	Use everyday objects as a reference to easily identify portion sizes of each food.
Vegetables	White rice and typical Puerto Rican beans can be part of a Mediterranean diet by decreasing the amount of rice (to 1/2 cup) and increasing the amount of beans (to a cup). Make sure you eat beans and not just stew broth!	The most consumed subgroups of vegetables in Puerto Rico are fruits (such as tomatoes), leaves (such as lettuce and spinach), flowers (such as broccoli and cauliflower), stems (such as asparagus), grains (such as green beans), pods (such as peas), bulbs (like onions), and roots (like carrots).
Fruits	Consume fruits of all colors and add more variety: green (grapes, kiwi), red (guava, strawberries), orange (papaya, melon, mango, grapefruit), white (pears, *carambola*), purple (grapes), and yellow (lemon).	A healthy eating pattern in Puerto Rico includes fruits such as *acerola*, passion fruit, pineapple, mango, oranges, grapefruit, papaya, soursop, plum, guava, melon, ripe banana, raisins, and cherries.
Meats	Limit the consumption of red meats (beef, pork, goat) and processed meats (ham, ham, sausage, salami, bacon, and other sausages).	Chicken and turkey are the most consumed poultry in Puerto Rico as part of a healthy eating pattern.
Fish	The typical fish of a Mediterranean diet that we consume in Puerto Rico include salmon, cod, tuna (can be canned in water), tilapia, sardines, and trout.	The most consumed fish in Puerto Rico are tuna, codfish, salmon, grouper, red snapper, sardines, tilapia, crab, shrimp, and clams.
Dairy products	When you select dairy, opt for low-fat (or fat-free) milk, natural cheese instead of processed, and Greek yogurt instead of high sugar. Avoid butter and margarine.	Milk and its derived products constitute essential sources of vitamins, minerals, and necessary proteins in our daily nutrition.
Fats	NA	The types of fatty acids are: monounsaturated (such as olive oil, canola, peanuts, and walnuts), polyunsaturated (such as corn oil, soy, and safflower), omega 6 (such as nuts and seeds), and omega 3 (as fish).
Alcohol	If you already drink alcohol, limit your consumption to 2 or fewer drinks per day if you are a man or 1 or fewer drinks per day if you are a woman. If you do not drink alcohol, you do not have to start doing it.	NA
Examples of healthy foods/drinks	NA	A healthy eating pattern in Puerto Rico includes non- or low-fat dairy products, such as milk, yogurt, cheese and fortified soy drinks.
Other meals (that is, spices, sugary drinks, desserts)	Limit consuming sugars by drinking less soda! Try drinking water instead of soda. Dilute juice in water.	NA

NA, Not applicable; PROMED, Puerto Rican Optimized Mediterranean-like Diet.

### *Retention*

Multiple strategies were implemented to increase retention in the study. First, participants were called up to five times for reminders of appointments. They were contacted by their preferred method of communication (email, text, phone, mail). Culturally-appropriate recruitment, retention, and engagement strategies were used based on previous studies on Puerto Ricans and similar Latino heritages, including showing familiarity, trust, and respect; obtaining at least three means of contact; sharing the participant’s clinical results in a simplified letter along with guidelines for clinically-acceptable values for consultation with their doctor; reimbursing transportation expenses or providing free transportation to/from FDI; providing snacks after each visit; allowing for flexible appointments (including weekends) and completion by phone; and using appropriate literacy level of materials and questionnaires [[98], [99], [100]]. During the 2019 earthquakes and the COVID-19 pandemic, participants received information on free or low-cost services, including mental health, and instructions for remaining in contact with the study. The value of their participation to scientific knowledge and future population health was also highlighted. These strategies benefited participants, even when there were no direct benefits from participating in the study.

### Statistical Analysis

Table 4 shows the minimum detectable effect sizes estimated for primary and secondary outcomes for the original sample size (n = 100) and adjusted sample size after COVID-19 protocols (n = 50), accounting for 10% loss-to-follow-up, using ANCOVA test [101] with 0.05 Type I error and 80% power.

**TABLE 4:** Minimum detectable effect sizes (4 mo means adjusted for baseline) for primary and secondary outcomes for PROMED, using ANCOVA test for 80% power and 5% Type I error rate

	Mean (SD) at baseline	Empirical Corr. b∖w baseline and 4 mo values	MDE for n = 90 (45 per arm)	MDE for n = 44 (22 per arm)
10-item cardiometabolic improvement score	5.4 ± 1.4	0.94	0.28	0.40
Weight (lbs.)	201.2 ± 42.3	0.97	6.07	8.68
Waist circumference (cm)	42.9 ± 4.9	0.92	1.13	1.62
BMI (kg/m^2^)	34.2 ± 6.6	0.86	1.99	2.84
Systolic BP (mmHg)	123.4 ± 17.1	0.80	6.06	8.66
Diastolic BP (mmHg)	80.4 ± 9.3	0.49	4.79	6.85
Total cholesterol (mg/dL)	196 ± 46.5	0.59	22.17	31.71
LDL-C (mg/dL)	123.4 ± 37.4	0.83	12.32	17.62
HDL-C (mg/dL)	48.9 ± 12.4	0.83	4.08	5.84
Triglycerides (mg/dL)	118.2 ± 73.0	0.74	28.99	41.47
Glucose (mg/dL)	101.6 ± 29.4	0.80	10.42	14.90
Hemoglobin A1c (%)	6.0 ± 1.3	0.77	0.49	0.70
Diet quality (score)	35.2 ± 3.7	0.85	1.15	1.65
Diet satisfaction (score)	94.9 ± 18.9	0.85	5.88	8.41

BP, blood pressure; PROMED, Puerto Rican Optimized Mediterranean-like Diet. ∗The results account for 10% loss-to-follow-up for original sample sizes of n = 100 and n = 50. ∗∗The minimum detectable effect sizes (MDE) by the ANCOVA approach is calculated based on $MDE=\frac{4{\left(Z_{1-\frac{\alpha }{2}}+Z_{\beta }\right)}^{2}\left(1-Corr^{2}\right)SD^{2}}{n}$, where n is the sample size (active arm + control arm) after accounting for loss-to-follow-up, Corr is the correlation coefficient between the baseline and 4 mo values, SD is the standard derivation for the baseline value, $Z_{\tau }$ is the $\tau^{th}$ lower-quantile of the standard normal distribution, α=0.05 is the type I error rate, and β=0.8 is the power.

The primary analysis is intention-to-treat. Data analysis will follow the Consolidated Standards of Reporting Trials (CONSORT) guidelines [102]. Flowcharts will include the number of participants at each stage, including screened, eligible, randomized, and followed-up. At the 4-mo follow-up, the mean cardiometabolic improvement score for participants in the intervention arm versus the control arm will be compared using a two-sample *t*-test. If randomization does not control for differences between the treatment and control groups on baseline characteristics, those differences will be statistically controlled for using multivariable regression modeling. Secondary analysis will compare the change in individual cardiometabolic risk factors and dietary and psychosocial factors between intervention and control groups using an ANCOVA test. Repeated measures analysis will test differences in 2- and 4-mo outcomes. Prespecified subgroup analyses will be conducted by sex-at-birth, BMI status, pre-existing cardiometabolic profile, COVID-19 period (pre- vs. at-pandemic), and adherence status. Missing data and loss to follow-up will be corrected. Two-sided 0.05 type I error threshold for statistical significance will be used.

## Protocol Management

### Study safety and data management

An Advisory Committee supervised the study design, implementation, and progress. Safety, confidentiality, and comfort of the participants and staff were safeguarded by asking research assistants to wear laboratory coats, gloves, and any other protective gear needed, using private examination rooms, deidentifying or coding samples and data, using secure data collection and storage protocols, and implementing checkpoints during the interview to assess participant’s wellbeing. Adverse events were monitored and reported to the senior investigator team and IRB offices, even if unrelated to study protocols. Any serious adverse condition was immediately referred to the resident clinical staff and/or participant’s health practitioner.

All data were collected using the real-time web-based electronic data capture tool, ‘Research Electronic Data Capture’ (REDCap), hosted at Harvard TH Chan School of Public Health [103]. REDCap separates identifiable and identifiable data using a secure coding system supervised by the team data analyst; all data are anonymized before analysis. The system runs automatic data quality checks through predefined validation parameters. The senior project personnel manage and distribute the data generated from the study with coinvestigators or external investigators observing federal and local policies on disseminating and sharing research results.

### Intervention fidelity and blinding

The research team implemented several strategies to ensure consistency and integrity of protocol implementation. Research assistants were trained on standard operating procedures, including uniform data collection and processing and questionnaire implementation. Instruments were tested and calibrated before and during the study timeline. Toledo Laboratory is a CLIA-certified laboratory that ran internal and external quality control checks daily; monitored assays, reagents, and equipment weekly or as needed; and ran duplicate analysis for 10% of the samples. Participants and all study personnel were thoroughly informed of the importance of observing treatment fidelity. The nutritionist ensured receipt of the text messages daily. Any departures from the protocols were discussed at the bi-weekly team meetings, and immediate corrections were done and monitored when needed.

Three nutritionists delivered the counseling sessions. The following strategies were used to increase fidelity of session implementation: *1*) using a standardized written curriculum and protocol; *2*) having the same senior nutritionist provide and supervise the training; *3*) holding simulated and actual sessions supervised by the senior nutritionist; and *4*) having noninterviewing nutritionists observe actual sessions led by other nutritionists. To monitor fidelity, the team logged the timing and the questions and answers documented during each session, which were subsequently discussed, maintaining blinding.

To assist with blinding, participants were informed that they would follow a healthy eating pattern without identifying the type of diet of each arm. Senior investigators and research assistants taking the measurements were blinded to the participant’s group allocation. Only the nutritionists were aware of group allocation as they provided the nutritional counseling and sent the text messages. The nutritional counseling sessions were conducted in a separate closed room to ensure confidentiality of treatment allocation, which was a different room from where research assistants conducted the measurement visits to prevent study personnel from interacting. Participants were asked not to talk about their diets to the staff, except the nutritionist. Information about randomization status was kept in a separate password-protected spreadsheet, and only the nutritionist had access to it. Biological samples were coded, and the laboratory personnel were blind to study group allocation. Contamination was avoided by excluding individuals with a household member, close relative, or friend already in the study; instructing participants to avoid sharing information about the study or their diet with others; and scheduling single visits (that is, 1 participant at a time) to prevent participants from speaking to each other at the waiting area.

### Process evaluation

The research team monitored internal study protocols, factors extraneous to the study that may have still influenced it, and participants’ feedback bi-weekly. Based on these, minor changes were implemented. For example, the cognitive ability of some interested individuals at the early stages of recruitment was questioned because of inconsistent answers or forgetfulness. A cognitive assessment tool (Montreal Cognitive Assessment) validated for this population [104] was added at the baseline visit before confirming eligibility and randomization (and administered to pending individuals with uncertain cognitive ability). The tool was brief and did not add time or effort burden to the participants. Participants’ feedback was generally positive throughout the study, and no other major changes were made to the protocols, except for COVID-19-related changes.

### The impact of the COVID-19 pandemic

Given the susceptibility of PR to natural and manmade events, like hurricanes, tropical storms, earthquakes, droughts, and sociopolitical disruptions, contingency plans were in place to pause or modify study protocols. On March 16th, 2020, the COVID-19 pandemic was officially declared in PR, interrupting all in-person interventions and research activities as the local government imposed a complete lockdown and other preventive measures. The research team immediately provided participants with culturally relevant, trustworthy information and resources on COVID-19. A weekly COVID-19 questionnaire was implemented to monitor signs and symptoms, testing, management, food access, and behavioral and psychosocial changes during the pandemic for the remaining participants between May and July 2020.

During the pandemic, all ongoing text-based messages continued to be delivered as programmed. However, all scheduled and pending follow-up visits for March–May 2020 were canceled. The time sensitivity of the protocol meant that several enrolled participants were lost during this period. Completion of the questionnaire resumed via phone or video call in May 2020. In June 2020, some restrictions were lifted, and some in-person activities were resumed. Clinic visits were limited to biological samples and clinical measurements following strict safety procedures; participants had the option of having a licensed nurse and research assistant take the measurements at home instead of attending the clinic. Interviews and nutritional counseling sessions were completed by phone or video chat.

Furthermore, a safe and effective protocol was implemented to receive stool samples and other intervention materials from the participant’s car. During this time, the number of times to contact a participant was increased to 10. All new recruitment was suspended and resumed in July 2020 by distributing flyers at safe community sites (that is, supermarkets, pharmacies, and parking lots) and posting on social media. The target sample size had to be adjusted from 100 to 50 participants given the COVID-19 disruptions impact on budget and timeline.

## Discussion

PROMED aimed to determine the efficacy of a Mediterranean-like diet that has been culturally tailored to Puerto Ricans using traditional foods and strategies for decreasing cardiometabolic risk factors. The result from this pilot trial may support observational evidence from cohorts of Puerto Rican adults showing that a MedDiet-like score reflective of typical Puerto Rican dietary preferences is associated with a better cardiometabolic profile. Specifically, a cross-sectional study among adults living in PR showed an adjusted odds ratio (95% CI) of 0.78 (0.63, 0.97) of having abdominal obesity per unit increment of the MedDiet score [22]. Furthermore, in a longitudinal cohort of Puerto Rican adults residing in the mainland US, a higher MedDiet-like score was associated with clinically meaningful and statistically significant 2-y decreases in waist circumference (−0.52 ± 0.26 cm); BMI (−0.23 ± 0.08 kg/m^2^); log-insulin (−0.06 ± 0.02 μIU/mL); log-HOMA-IR (−0.05 ± 0.02), and log-CRP (−0.13 ± 0.03 mg/L) [21].

The MedDiet has been previously adapted and implemented among US-residing Latino adults, but not for Puerto Ricans, who are often grouped in the literature as of Latino ethnic heritage. For example, EnForma was a 3-mo program with no control arm that promoted a MedDiet-style dietary pattern focused on consuming typical Latino foods with high-quality dietary fats (both plant-based and fish) and high-quality carbohydrates [105] Nutritional counseling was given during two face-to-face sessions and two telephone sessions. No specific foods were provided or emphasized. The intervention also recommended increasing physical activity. The main outcome was acceptability, with lifestyle behavior changes at a 3-mo follow-up also reported. Of the 36 participants, 33 completed the program; most were of Mexican heritage, and the majority deemed the intervention helpful and acceptable. Modest improvements were noticed for mean dietary fat quality score [0.5 (95% CI: 0.0, 1.1)] and mean fruit-vegetable servings/d [0.7 (95% CI: 0.1, 1.3)]. EnForma was adapted from the Heart Healthy Lenoir Project, a 2-y randomized controlled trial focused on a Mediterranean-style dietary pattern and increased walking for predominantly African American adults [106]; adaptations included language, use of bilingual/bicultural staff, and culturally-relevant illustrations. EnForma was later translated into EnForma-Diabetes, a 2-mo intervention with similar protocols and outcomes targeting older Latinos with T2D [107]. The program enrolled 21 participants of predominantly Mexican descent and showed high engagement and acceptability and a significant increase in MedDiet score (2.3; 95% CI: 1.0, 3.5).

These studies suggest that MedDiet might be feasible and acceptable to the Latino population. However, the literature is limited to these few examples; these programs were short-term and had a narrow scope. First, the studies did not include a comparison group. Latinos generally respond well to nutrition education programs [108, 109] so it remains unknown if a MedDiet-focused program would be better than general healthy eating messages. Also, the programs were educational only, and nutrition education programs are more effective when coupled with behavioral skills, are based on prior research, or include individual, social and environmental changes; information alone is rarely sufficient to produce behavioral and health changes [110]. PREDIMED provided higher intensity of educational efforts to the intervention group than the control group, yet found that the observed changes in primary outcomes remained the same after accounting for this increased effort [111]. Another shortcoming of the existing programs is that they primarily focus on surface-level cultural adaptations (such as language and use of bilingual staff) rather than structural and deep-structure tailoring that account for the multilayered cultural attitudes and beliefs of the population and which are needed for more effective cultural sensitivity and appropriateness [89, 90, 112]. This is particularly important for Latinos, a diverse group of several ethnic heritages including Puerto Ricans, each with unique food preferences and health-related attitudes [[113], [114], [115]].

PROMED closes several of the existing gaps in the literature. The study included a control group consisting of the healthy eating advice currently used in PR, applied deep-structure cultural components, and provided familiar, accessible, and inexpensive preferred foods. Efficacy studies that typically provide the test food can mitigate structural barriers and add intensity to the intervention [116]. Furthermore, biological outcomes were measured to test the efficacy of the intervention not only on changes in dietary behaviors but also on cardiometabolic outcomes of importance to the PR population, which amplifies the potential for dissemination and implementation. A systematic review of cultural adaptations of the Diabetes Prevention Program found that most of them assessed adoption or feasibility only [117]. The adaptations made to PROMED from the evidence-based PREDIMED are thus expected to be culturally appropriate, acceptable, accessible, and feasible to follow for adults in PR.

PROMED had multiple strengths. First, the study was done in a free-living population, allowing participants freedom of food choices, mealtimes, and frequency of consumption. This approach likens PROMED to a pragmatic clinical trial [118]. Participants were not prescribed a rigid diet and were told they could incorporate the recommendations at their own pace to allow for flexibility within a more real-world setting. The intervention included individual in-person counseling reinforced through subsequent text messages rather than continued educational sessions, deemed less likely to be effective for this population and encompass barriers in time, childcare, and transportation [88, 119]. Text-based interventions were easy to deliver and have proven feasible, acceptable, and efficacious in other interventions for similar Latino groups [92, 120, 121]. Sending texts proved valuable during the COVID-19 pandemic, as the team could continue the intervention without interruptions. Another strength was including deep-structure cultural components that addressed PR-residing adults’ preferences and attitudes toward foods and eating behaviors. Furthermore, PROMED eased structural barriers of access, cost, and familiarity, increasing the likelihood of sustainability of the intervention.

Some limitations were identified in the methodology of PROMED. First, although several measures were taken to safeguard the blinding of outcome assessment, it cannot be guaranteed, given the nature of the study. Only the nutritionists providing the nutritional counseling were aware of group allocation; they did not perform any other study protocol or communicate with other personnel about the participants’ status. However, participants might inadvertently talk about their diet with the staff. Participants familiar with the PR nutritional guidelines or the MedDiet could have deduced their treatment allocation; however, the study information did not specify that it was contrasting these two diets but rather that participants would follow a healthy eating pattern. Another limitation was the COVID-19 delays and changes in protocols. The study lost several participants midstudy, and recruitment of new participants had to be limited, decreasing the final sample size considerably. The timing of the intervention was short, and although changes in blood pressure and lipid markers may be feasible with a healthy diet within 4 mo, weight changes are less salient [106, 122, 123]. The short timing and sample size also prevented from calculating the risk of developing endpoint disease (that is, T2D, hypertension, heart disease outcomes). Longer and larger interventions will be planned to determine if PROMED can be sustained and produce long-term benefits. Additionally, future research should engage community-based participatory research and implementation sciences approaches to strengthen outreach and dissemination of PROMED.

Adherence to a dietary intervention is an important—yet challenging—determinant of its effectiveness [124]. The team applied strategies to increase adherence to the dietary advice and to the food given to the intervention group, such as daily text reminders, takeaway visual aids, underscoring recommended intake amounts, and suggesting recipes or meals to incorporate the food provided. The cognitive function screener also helped exclude individuals with a low likelihood of compliance. The dietary intake questionnaires will allow gauging adherence at the study conclusion. Still, real-time adherence assessments were not included (such as observing participants’ food consumption, collecting daily dietary records, or weighing leftover food), given the short study timeline and its focus on free-living individuals in a real-world setting. Thus, adherence to PROMED would reflect how well individuals comply with dietary advice and recommended foods. Still, to better assess adherence, future studies should consider adding more frequent contact with the nutritionist, collecting periodic detailed dietary records, using supportive aids (such as measuring tools), and collecting objective biomarkers of intake [116, 124]. Further, multidimensional constructs of adherence, that is, behavioral and dietary adherence, may be considered for more accuracy [125]. The stored biospecimens may allow to measure metabolic signatures of MedDiet as objective biomarkers of adherence in the future [126].

The results of this pilot study will support planning and implementing more extensive intervention trials that address the current limitations and help boost the evidence on the healthfulness of a culturally-appropriate MedDiet. If PROMED is proven efficacious in improving cardiometabolic risk factors, dietary intake, and behaviors, the findings may be applied to broader disease-prevention programs and policies in PR. For example, by incorporating PROMED modules into the current healthy eating materials and clinical programs provided by nutritionists in PR; providing or subsiding healthy foods as a replacement for unhealthy ones in government or community food programs; creating incentives for local crops (that is, beans) or other products of superior nutritional value; expanding health insurance coverage of PROMED (or similar nutrition programs) in clinical settings; and [5] developing population-wide health promotion strategies to increase knowledge on how to follow an optimized MedDiet-like tailored to Puerto Ricans.

## Author Contribution

JM designed the research, supervised and validated study implementation, created data visualization, wrote the manuscript, and had primary responsibility for final content. CBDA contributed to study design, conducted research, created data visualization, and wrote portions of the manuscript. CA and FGV contributed to study design and provided essential materials. JON: contributed to study design, created data visualization, and wrote portions of the manuscript. CG: contributed to analytical plan and performed statistical analyses for power calculations. DS: performed statistical analyses for power calculations and contributed to study design and discussion. CFRB, VM, FBH, and WCW contributed to study design and discussion. JFRO provided essential resources and materials, supervised and validated study implementation, and contributed to study design and discussion. All authors have read and approved the final manuscript. Data described in the manuscript, code book, and analytic code will be made available upon request.

## Data Availability

Data described in the manuscript, code book, and analytic code will be made available upon request pending application and approval to the corresponding author.

## Funding

This work was supported by anonymous private donations and the Rita Allen Foundation. Statistical advice and technical assistance were provided by Harvard Catalyst: The Harvard Clinical and Translational Science Center (NCATS-NIH Award UL1 TR002541). The funding sources had no role in the design or execution of this study and will not have any role during its analyses, interpretation of the data, or decision to submit results. This study was funded through an anonymous foundation award and institutional funds.

## Author disclosures

The authors report no conflicts of interest.

## Appendix A Supplementary data

Multimedia components 1 Multimedia components 1
