# Supplementary material for: Design and Implementation of a Culturally-Tailored Randomized Pilot Trial: Puerto Rican Optimized Mediterranean-Like Diet
Source: Curr Dev Nutr. 2022 Dec 23;7(1):100022. doi: 10.1016/j.cdnut.2022.100022 (PMC10100940; doi:10.1016/j.cdnut.2022.100022)
Supplement: Multimedia components 1 [file mmc1.docx]

Design and Implementation of a Culturally-Tailored Randomized Pilot Trial: Puerto Rican Optimized Mediterranean-like Diet (PROMED)

Mattei *et al*.

Supplemental Figure 1. Visual guide for the PROMED control group

Source: Guidelines for Healthy Eating and Physical Activity for Puerto Rico. Comisión de Alimentación y Nutrición de Puerto Rico. College of Nutritionists and Dietitians of Puerto Rico. 2015. https://nutricionpr.org/wp-content/uploads/2018/02/GuiaAlimentaria.pdf

Supplemental Figure 2. Visual guide for the PROMED intervention group
